# Transmission of Diverse Variants of Strawberry Viruses Is Governed by a Vector Species

**DOI:** 10.3390/v14071362

**Published:** 2022-06-23

**Authors:** Igor Koloniuk, Alena Matyášová, Sára Brázdová, Jana Veselá, Jaroslava Přibylová, Jana Fránová, Santiago F. Elena

**Affiliations:** 1 Institute of Plant Molecular Biology, Department of Plant Virology, Biology Centre CAS, 370 05 Ceske Budejovice, Czech Republic; matyasova@umbr.cas.cz (A.M.); sara.souckova@umbr.cas.cz (S.B.); jana.vesela@umbr.cas.cz (J.V.); pribyl@umbr.cas.cz (J.P.); jana@umbr.cas.cz (J.F.); 2 Faculty of Agriculture, University of South Bohemia, 370 05 Ceske Budejovice, Czech Republic; 3 Instituto de Biología Integrativa de Sistemas, CSIC-Universitat de València, Paterna, 46980 València, Spain; santiago.elena@csic.es; 4 Santa Fe Institute, Santa Fe, NM 87501, USA

**Keywords:** aphid transmission, multiple infections, plant virus, strawberry virus

## Abstract

Advances in high-throughput sequencing methods have boosted the discovery of multistrain viral infections in diverse plant systems. This phenomenon appears to be pervasive for certain viral species. However, our knowledge of the transmission aspects leading to the establishment of such mixed infections is limited. Recently, we reported a mixed infection of a single strawberry plant with strawberry mottle virus (SMoV), strawberry crinkle virus (SCV) and strawberry virus 1 (StrV-1). While SCV and StrV-1 are represented by two and three molecular variants, respectively, SmoV has three different RNA1 and RNA2 segments. In this study, we focus on virus acquisition by individual adult aphids of the *Aphis gossypii*, *Aphis forbesi* and *Chaetosiphon fragaefolii* species. Single-aphid transmission trials are performed under experimental conditions. Both different viruses and individual virus strains show varying performances in single aphid acquisition. The obtained data suggests that numerous individual transmission events lead to the establishment of multistrain infections. These data will be important for the development of epidemiological models in plant virology.

## 1. Introduction

Plant viruses enlist a diverse group of viral species that often rely on transmission via invertebrate vectors [1]. While for annual plants, viral infections are limited by the rather short lifespan of the host, perennial plant species have increased chances of being recurrently infected. The wide adoption and affordability of high-throughput sequencing (HTS) has led to the discovery of numerous novel viruses as well as provided an indispensable tool for the in-depth characterization of viromes at the level of a single host individual. Although it was suggested that we have limited knowledge about the size and diversity of viral families [2], special cases include so-called ecogenomics virus studies, when a single host virome is the object of the study [2,3,4]. Such viromes may be represented by single infections as well as by more complex situations with multiple viral species, sometimes represented by several isolates, concurrently infecting the same host [3,4,5], which should be clearly differentiated from viral quasispecies or mutant clouds generated during the error-prone replication of RNA genomes [6,7,8]. These multi-isolate, or multi-strain, viral infections appear to be common [9,10]. For mixed virus infections, both antagonistic and synergistic interactions have been described [5], but strains of the same species might be under greater pressure as they exploit the same replication pathways. Interestingly, antagonistic interactions of strains were applied for plant ‘vaccination’ purposes when plants infected by a mild viral strain were protected from infection with an aggressive one, a phenomenon called ‘cross-protection’ [11,12,13]. Based on a similar principle, genetically modified crops expressing viral capsid proteins were developed to introduce resistance to related viruses [14].

So far, our current knowledge about multistrain infections is based on studies of citrus tristeza virus (CTV; *Closteroviridae*) [11,12,15] and potato virus Y (PVY; *Potyviridae*) [16,17]. Although infections with more than one strain, or genotype, in a single host organism have been known for some time, recent studies have documented a growing number of cases [9,10].

Modes of arthropod-mediated transmission of plant viruses might be non-persistent or semi–persistent [1,18]. Upon that, a vector is infectious for a time that varies from minutes to many hours. In contrast, during circulative transmission, a vector remains infectious for its entire lifespan and requires a longer acquisition time that is followed by virus circulation in the vector’s organism [19]. Lastly, propagative circulative transmission is hallmarked by a virus’s ability to replicate in a vector, which serves both as a host and a vector [19,20,21].

Nevertheless, the establishment of a viral infection in a susceptible host must overcome numerous barriers [19,22,23]. During propagative transmission, salivary glands represent one of those barriers [24]. Such constraints create a bottleneck for a viral population during vector-mediated transmission. For viruses transmitted in a non-circulative manner, a limited number of studies focused on the estimation of the absolute number of virions transferred to a host during its inoculation by a vector. For PVY, the number was assessed to be extremely low—several particles per aphid [25]. A decreased number of original artificially produced viral variants of cucumber mosaic virus (*Bromoviridae*), a non-persistent virus with a tripartite genome, were transmitted by aphids [21,26].

Nearly 30 different viruses have been reported to infect strawberries, which are predominantly transmitted by different aphid species [27,28,29,30,31]. It is recognized that yield losses are affected by frequently observed mixed viral infections [32,33]. Both strawberry crinkle virus (SCV; genus *Cytorhabdovirus*, family *Rhabdoviridae*, negative-sense ssRNA genome) and strawberry mottle virus (SMoV genus *Stramovirus*, family *Secoviridae*, bipartite positive-sense ssRNA genome) are strawberry viruses known for decades [27,32], while strawberry virus 1 (StrV-1; genus *Cytorhabdovirus*) was reported in China and in the Czech Republic in 2019 [29,34]. All three viruses were shown to be transmitted by aphid vectors [27]. While StrV-1 still lacks any data on its biological relevance, SCV and SMoV are among the most important and widely spread viruses infecting strawberries and contribute to strawberry decline disease [27]. The symptoms’ severity depends both on the strawberry cultivars and the species composition of the infecting viruses [27,35].

In this study, we examined the transmission peculiarities of three different viral species, SCV, SMoV and StrV-1, and their genetic variants (genotypes) previously reported to infect a single strawberry, *Fragaria × ananassa* (Weston) Duchesne plant [29,36]. Both graft- and single-aphid-mediated transmission experiments were conducted. Then, we evaluated intraspecies cross-protection between variants of SMoV and StrV-1. Further, we investigated the prevalence of the viruses and their variants in individual aphids.

## 2. Materials and Methods

### 2.1. Plant Materials

The CRM3 isolate of *F. ananassa*, cultivar Cacanska rana, was used as a virus source. The CRM3 clones, or daughter plants, were produced using stolons. The CRM3 plant was reported previously to be infected with the following three viruses: SCV, SMoV and StrV-1 [29,36]. Each virus was represented with several molecular strains (variants, or genotypes). The variants were arbitrarily named as A, B and C, if applicable, as follows: SCV–SCV^A^, SCV^B^; SMoV–SMoV RNA1^A^, RNA1^B^, RNA1^C^, RNA2^A^, RNA2^B^, and RNA2^C^; StrV-1–StrV-1^A^, StrV-1^B^, StrV-1^C^ [29,36]. Note that any correlation between names of RNA1s and RNA2s of SMoV, i.e., RNA1^A^–RNA2^A^ was not investigated. The plants were maintained in a custom-built mesh cage. Recipient plants were Alpine strawberry, *Fragaria vesca* L. *v.* f. *semperflorens,* raised from seeds. All plants were maintained in an insect-proof greenhouse with a controlled temperature and a 16-h day/8-h night period.

### 2.2. Aphid Cultures

The following three aphid species were used in this work: the strawberry root aphid *Aphis forbesi* Weed, the melon aphid *Aphis gossypii* Glover and the strawberry aphid *Chaetosiphon fragaefolii* Cockerell. Isoclonal aphid cultures were started from the field-collected aphids. A single 1st instar nymph was isolated and transferred to a new *F. vesca* plant. This step was repeated three times. The colonies were maintained by transferring unwinged adult individuals to a new *F. vesca* plants every month.

Their taxonomic identification was performed using molecular barcoding (*cox* and *cytb* genes, Table S1) with published primers [37,38] and Phire Tissue Direct PCR Master Mix following the manufacturer’s recommendations (Thermo Scientific, Waltham, MA, USA). The partial *cox* gene sequences were deposited at the NCBI repository under accession numbers OK181865.1, ON756037 and MN420510.1 for *A. gossypii*, *A. forbesi* and *C. fragaefolii*, respectively. The partial *cytb* gene sequences have accession numbers ON756035, ON756036, and ON756034 for *A. gossypii*, *A. forbesi* and *C. fragaefolii*, respectively.

For aphid-mediated transmission experiments, ten adult aphids were transferred to a CRM3 clonal plant and eight weeks later were used for the transmission and virus detection assays.

### 2.3. Transmission Assays

For each transmission, a wingless aphid adult from a CRM3 plant’s colony was transferred using a fine paintbrush on a leaf of a recipient plant that was placed in the mesh cage until the aphid’s removal. Adults were allowed to oviposit on virus-free Alpine strawberry plants for 24 h. After that, the plants were sprayed with an insecticide, FAST M (active substance: deltamethrin 0.12 g/L) and transferred to the greenhouse at 25 °C 16-h/8-h day. The plant samples were collected and analyzed thirty days post inoculation.

Graft-mediated virus transmission was performed via petiole wedge grafting technique using two trifoliate leaves of the CRM3 plant per a seed-raised Alpine strawberry plant. The plant samples were collected and analyzed sixty days post grafting.

### 2.4. RNA Isolation

Total plant RNA extraction was performed from 50 mg of plant fresh material snap-frozen in liquid nitrogen using the Thermo Scientific GeneJET Plant RNA Purification Mini Kit (Thermo Scientific) following the manufacturers’ recommendations. Total aphid RNA extraction was performed using TRI Reagent (Sigma Aldrich, St. Louis, MO, USA) or TRIzol (Invitrogen, Carlsbad, CA, USA) kits from a single aphid individual. Quantification and quality control of RNA extracts were performed using Nanodrop 1000 UV-Vis spectrophotometer and Qubit HS RNA and IQ assays (Invitrogen).

### 2.5. cDNA Synthesis

Total RNA (50–100 ng of total aphid RNA and 200–400 ng of total plant RNA) was reverse transcribed in cDNA using Maxima H Minus First Strand cDNA Synthesis Kit with dsDNase (Thermo Scientific) in 20 µL reactions following manufacturers’ recommendations. The cDNA was then diluted 1:10 with milliQ-grade water and proceeded for qPCR assays. Each reaction was run in duplicate. No template controls for all primer sets and no reverse transcriptase controls for the aphids’ endogenous transcripts (Table S1) were included.

### 2.6. Genotype-Specific RT-qPCR

RT-qPCR assays were conducted on the CFX96 real-time PCR detection system (Bio-Rad, Hercules, CA, USA). The 10 µL reaction was prepared from 5 µL of tenfold diluted cDNA, 0.25 µL of forward and reverse primers (10 mM, final concentration 250 nM, Table S1), 2.75 μL of nuclease-free water and 2 µL of 5 × HOT FIREPol EvaGreen qPCR Mix Plus (Solis BioDyne, Taru, Estonia).

The reaction conditions were set up with three-step cycling protocol—95 °C for 12 min, followed by 40 cycles of 95 °C for 10 s, 60 °C for 20 s and 72 °C for 20 s. Dissociation curve analysis was performed by ramping from 65 °C to 95 °C (with increments of 0.5 °C for 5 s) to verify the specificity of primer amplification and the presence of potential primer dimers based on the presence of a single peak. No template, positive, and, if necessary, no reverse transcriptase controls were included to check the potential cross-contamination and presence of genomic DNA. As an internal endogenous control for levels of plant material presence, the NADH mRNA was used (Table S1, [39]). Amplification efficiency (E, Table S1) and correlation coefficient (R2, Table S1) were tested by a standard curve based on serial dilutions of the cDNA template. The data were analyzed using Bio-Rad CFX Maestro 1.1 (Bio-Rad) and R software version 4.1.0 [40] under Rstudio version 2021.09.2+382.

### 2.7. Determination of Ratios between Viral RNA Strands

Total RNA was reverse-transcribed with virus-specific primer (Table S1) and 1:10 diluted cDNA preparation was used for qPCR. Virus-specific primers hybridized to N and P3 gene regions (i.e., adjacent to the *P* gene, where the qPCR primers target, Figure S1). These primers were selected to avoid the overestimation of positive-strand values as there are mRNA molecules transcribed from the *P* gene in addition to the antigenomic positive RNA strand.

As nearly all the aphids previously showed positivity in qPCR, generic cDNA primers, i.e., those targeting all the virus variants and the variant-specific primers (Table S1), were used for qPCR assays.

The obtained *C_q_* values were used to calculate the ratio between negative and positive strands of viral RNAs using the formula $ratio=efficiency^{\left(\Delta C_{q}^{positive\;strand}-\Delta C_{q}^{negative\;strand}\right)}$, with the experimentally calculated efficiency of the corresponding primers (Table S1, SCV^A^—1.98, SCV^B^—1.95, StrV-1^A^—2.01, StrV-1^B^—1.96).

### 2.8. High-Throughput Sequencing

The original CRM3 *F. ananassa* and the *F. vesca* cv. Alpine strawberry plant 4 from the graft-transmission experiment were analyzed. Non-degenerate primers were designed in conserved regions (Table S1) and a series of PCRs using Phusion Flash High-Fidelity PCR Master Mix (Thermo Scientific) were performed to amplify nearly full-length sequences of viral genomes (Figure S2). Sequencing libraries were prepared from equimolarly mixed PCR amplicons that were priorly purified with Agencourt AMPure XP beads (Beckman, Brea, CA, USA) following the manufacturer’s recommendations. The NEBNext Ultra™ II FS DNA Library Prep Kit for Illumina was used in combination with NEBNext Multiplex Oligos (NEB, Ipswich, MA, USA) to prepare sequencing libraries that were processed by Illumina NovaSeq 6000 in 200 bp paired-end mode. The obtained data were deposited at the NCBI repository under accession numbers SAMN27687906-8.

### 2.9. Data Analysis

All sequence data were analyzed using Geneious 2020 (Biomatters, Auckland, New Zealand) and CLC Genomics Workbench 9.5.1 (Qiagen, Hilden, Germany). All RT-qPCR data were processed with Bio-Rad CFX Maestro 1.1, version 4.1 (Bio-Rad), and further data analyses were conducted in R version 4.1.0 [40] and the ggplot2 package version 3.3.5 [41] under RStudio version 2021.09.2+382. All data are available in the Supplementary Materials.

## 3. Results

### 3.1. Stability of Genomic Sequences of Individual Variants

To assess the RT-qPCR results, we performed recombination analyses for each virus. Such events might jeopardize virus variant identification that was based on a single region. A series of PCRs were performed to amplify nearly full-length sequences of viral genomes that were deeply sequenced on the Illumina platform.

The data analysis involved several mapping steps. In order to filter out non-chimeric viral sequences, stringent mapping of quality and adapter-trimmed reads to viral references was performed (97% identity for 100 nt overlap with 97% overall identity of mapped read cut-off values were applied in the CLC mapping tool). Between 81% and 90% of the reads were totally mapped (Table S2), which originated from so-called master viral sequences and their mutant clouds. Subsequently, all unmapped reads were collected and re-aligned against the viral references with relaxed conditions (97% of identity for 50 nt overlap with 60% overall identity of mapped read cut-off values). Only a small fraction of previously unmapped reads matched viral references (0.2–11.6%). Revealed variation sites were mainly of a single nucleotide polymorphism, but no chimeric/recombinant or structural variants were detected (Tables S3 and S4). Although gel electrophoresis of the PCR products (Figure S2) showed the presence of bands smaller than viral genomic segments, de novo assembly produced several contigs that were identified as plant ribosomal RNAs (not shown).

### 3.2. Viruses and Their Variants Were Present in All Leaves, Leaf Parts and Runners

In order to estimate the plant-wide distribution of viruses and their variants, the following CRM3 plant samples were collected: young and old trifoliate leaves from the crown part, and the short stem, leaves of the daughter plant and terminal leaves from runners. All three viruses, including their variants, were detected.

### 3.3. Graft Transmission

**Figure 1:**
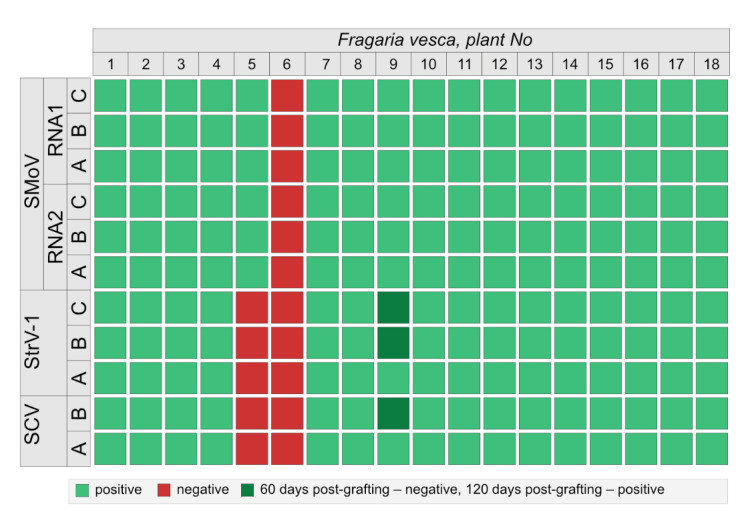
Virus testing for SCV, SMoV and StrV-1 and their variants following grafting of the CRM3 plant to 16 *F. vesca* recipient plants after 30 dpg; plants 5, 6 and 9 were additionally tested 60 dpg.

Two trifoliate leaves of the CRM3 plant were graft-transmitted to each of the eighteen recipient *F. vesca* plants. Sixty days post-grafting (dpg), one plant was tested as virus-free, one was infected only with SMoV, one with SMoV and some of the SCV and StrV-1 variants (Figure 1), while the remaining 13 (81%) tested positive for three viruses, including all variants.

Three of these plants were retested at 120 dpg with confirmation of the negative and the SMoV results. For plant 9, all the variants of SCV and StrV-1 were detected, respectively (Figure 1). Thus, complete transmission of all three viruses and all their variants was successful in 14 out of 16 plants (88%). Symptomatic infection was observed in all SMoV-positive plants, but not in the cases of virus-negative or positive for SCV and StrV-1 plants, such as plants 6 and 5, respectively.

### 3.4. Absence of Cross-Protection between StrV-1^A^ and ^B^ Variants

Two trifoliate leaves of the *F. vesca* plant infected with either the A or B variant of StrV-1 were separately graft-transmitted to *F. vesca* plants (*n* = 3) and tested at 30 dpg. After confirmation of the infection, the plants were used for graft transmission with the other variant (i.e., the StrV-1^A^-positive plant was grafted with the StrV-1^B^ material and vice versa). Both StrV-1^A^ and ^B^ were detected in the grafted plants at 30 dpg.

### 3.5. Absence of Cross-Protection between SMoV Variants

Two leaves of an *F. vesca* plant infected with either RNA1^A^:RNA2^C^ or RNA1^BC^:RNA2^AC^ of SMoV were separately graft-transmitted to *F. vesca* plants (*n* = 3) and tested at 30 dpg for the virus variants. After confirmation of the infection, the plants were used for the second graft transmission with the opposite variant and were tested at 30 dpg. The plants initially infected with RNA1^A^:RNA2^C^ were not protected against SMoV variants RNA1^BC^ or RNA2^A^ and, otherwise, RNA1^BC^:RNA2^AC^ plants were subsequently infected with RNA1^A^. A similar experiment with plants (*n* = 3) initially infected with SMoV RNA1^BC^:RNA2^AC^ resulted in successful infection with RNA1^A^:RNA2^B^ by graft transmission from the SMoV RNA1^ABC^:RNA2^ABC^ donor.

### 3.6. Virus Detection in Aphids

**Figure 2:**
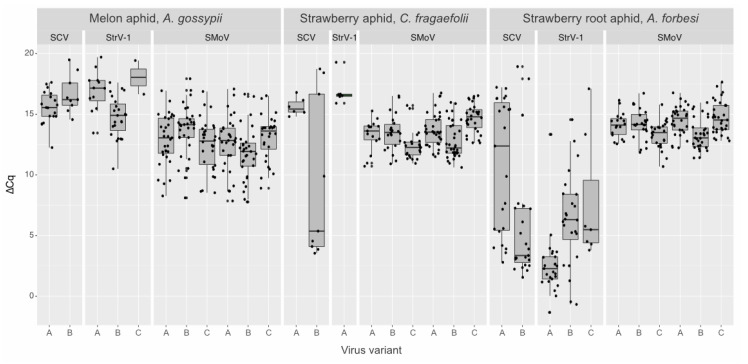
Boxplot distribution of ∆*C_q_* values of SCV, SMoV and StrV-1 and their variants in three aphid species. Individual ∆*C_q_* values are overplotted as points.

We established separate aphid colonies on the daughter CRM3 plants and, after 60 days, tested individual adult wingless aphids of *A. forbesi*, *A. gossypii* and *C. fragaefolii*. As the endogenous control, aphids’ 16S RNA was employed and its *C_q_* was subtracted from the *C_q_* values of viral targets. The obtained data showed a broad range of the ∆*C_q_* values for rhabdoviruses—from −1 to 20 (Figure 2). As an internal control for plant material, the NADH mRNA was used, which was negative in all aphid samples, i.e., no *C_q_* value was produced. Notably, some ∆*C_q_* values for rhabdoviruses, especially from *A. gossypii* and *C. fragaefolii*, were comparable with those of SMoV (Figure 2).

**Figure 3:**
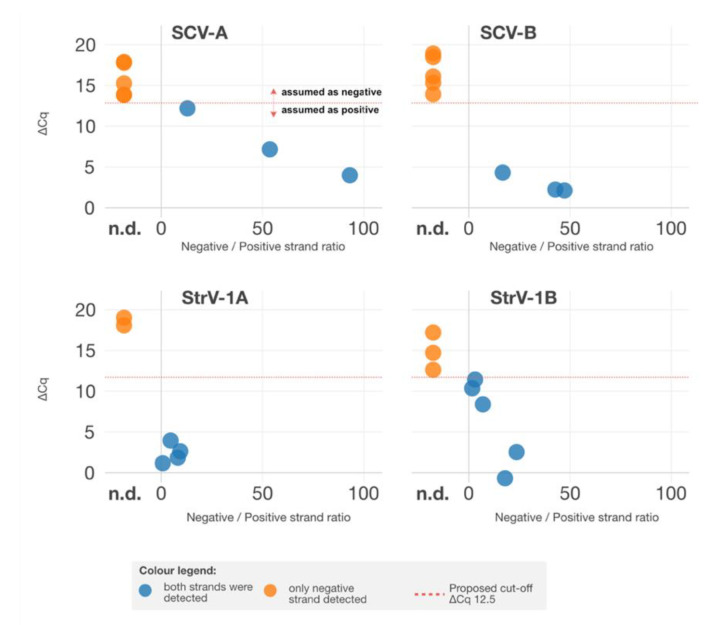
Comparison of ∆*C_q_* values against ratios between negative and positive strands. Cases when only one, negative, strand was detected are annotated by an orange color and were aligned against the n.d. (not detected) sign on the abscissa axis.

Therefore, to verify whether there was ongoing rhabdoviral replication and, subsequently, both viral genomic (negative) and antigenomic (positive) strands should be present, we estimated the ratios between negative and positive strands and compared the ratios against the corresponding ∆*C_q_* values (Figure 3) in eight samples. Interestingly, only a negative strand representing rhabdoviral genomic RNA was detected for cases with ∆*C_q_* > 12.5, while different ratios of negative and antigenomic positive strands were obtained for the remaining cases.

**Figure 4:**
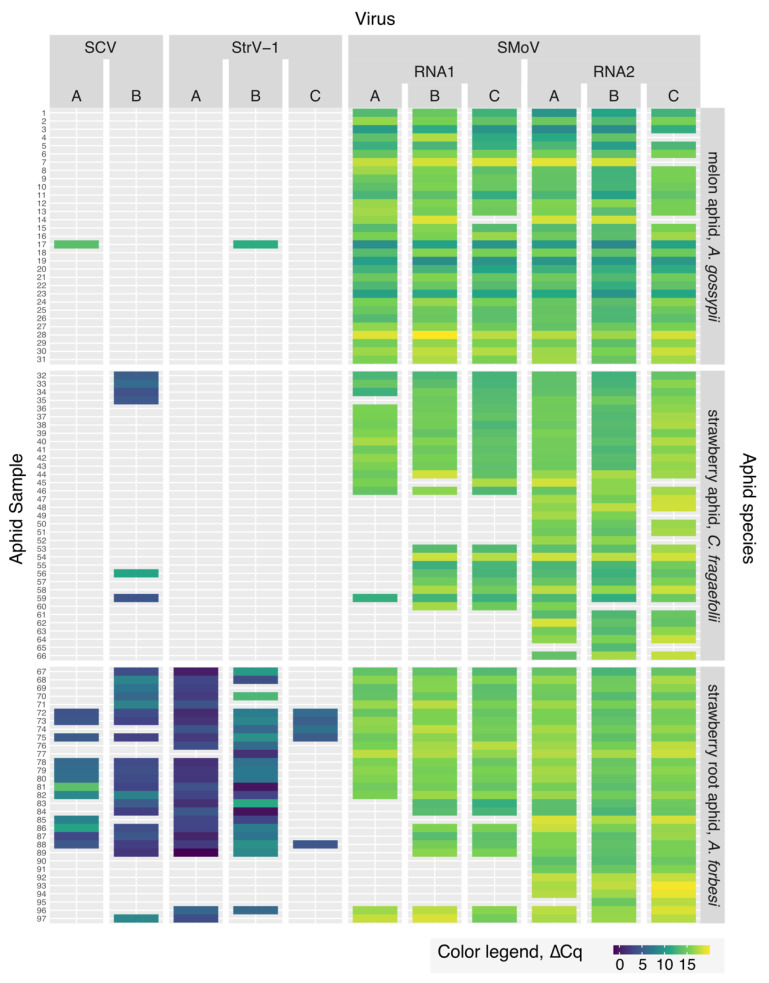
Overview of ∆*C_q_* color-coded values for SCV, SMoV, StrV-1 and their variants in individual aphid adults for three species—*A. forbesi* (*n* = 31), *A. gossypii* (*n* = 31) and *C. fragaefolii* (*n* = 35). Each row represents a single aphid. Refer to Table S5 for unfiltered data.

Using the calculated ∆*C_q_* = 12.5 as a cutoff, the majority of SCV and StrV-1 values for *A. gossypii* and *C. fragaefolii* were filtered out (Figure 4; the unfiltered values are available in Table S5).

The most striking differences between aphid species were observed in the case of rhabdoviral species, SCV and StrV-1. *A. forbesi* showed the highest fraction of either SCV- or StrV-1-positive individuals (81%) in comparison to *A. gossypii* and *C. fragaefolii* (3% and 17%, respectively). Furthermore, only *A. forbesi* individuals contained several variants of either SCV and/or StrV-1, while only one *A. gossypii* individual (#17, Figure 4) had a variant of SCV and StrV-1. For *A. gossypii*, all six animals had the same StrV-1^B^ variant. Additionally, note the lower fraction of *A. forbesi* aphids with the StrV-1^C^ in comparison with the StrV-1^A^ and StrV-1^B^ variants.

The distribution of SMoV variants in *A. forbesi*, *A. gossypii* and *C. fragaefolii* individuals varied widely. The observed differences were found between species, with *C. fragaefolii* having the lowest percentages of virus-positive individuals (Table 1).

Notably, there were *A. forbesi* and *C. fragaefolii* individuals in which only RNA2 of SMoV was detected (Figure 4, Table 1).

### 3.7. Statistical Analyses of Presence of Viruses and Their Variants in the Three Aphid Species

To gain additional quantitative insights into the prevalence of the different viruses and variants in the three aphid species, we fitted the data in Table 1 to the following binomial logistic regression model: $\mathrm{logit}\left(F\right)\sim \phi +A+V+R\left(V\right)+A\times V+A\times R\left(V\right)+\varepsilon_{ijkl}$, where *F* stands for the frequency of viral variant *R* from virus species *V* detected in aphid species *A*, *φ* corresponds to the expected grand mean of *F* and *ε* represents the error term assumed to be binomial. The model incorporates aphid and viral species as orthogonal factors and the RNA variant as a nested factor within the viral species. Finally, a logit link function was chosen. Significant effects were only found to be associated with aphid species (*χ*^2^ = 68.79, 2 d.f., *p* < 0.001) and with viral species (*χ*^2^ = 610.99, 2 d.f., *p* < 0.001). On average, the viral prevalence was higher in *A. forbesi* (68.6%) and relatively similar and lower for *A. gossypii* (33.3%) and *C. fragaefolii*, respectively. Among viruses, the highest prevalence was found for SMoV (94.5%), and it was much lower and similar for the two cytorhabdoviruses (17.2% for SCV and 18.0% for StrV-1). Interestingly, the interaction term between virus and aphid species was not significant (*χ*^2^ = 0, 4 d.f., *p* = 1), suggesting that the observed differences among aphids were independent of the virus being analyzed.

**Figure 5:**
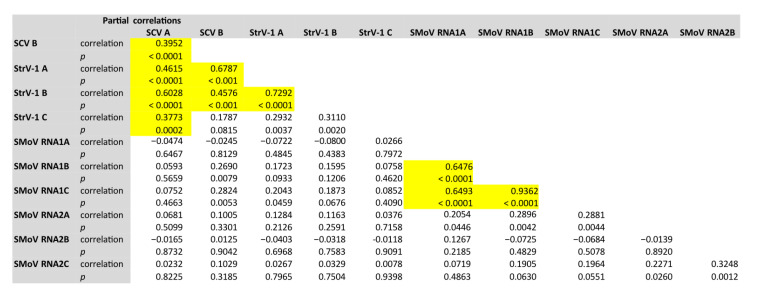
Pairwise partial correlations. Significant cases after applying the false discovery rate correction are highlighted in yellow. In all tests, the number of degrees of freedom was 94.

Next, we sought to evaluate whether the presence or not of a given viral variant was dependent upon the presence of other variants of the same or another virus. To do so, we computed pairwise partial (controlling for aphid species) correlation coefficients and applied a stringent false discovery rate correction to the *p*-values to account for multiple comparisons of the same null hypothesis of no association between viruses. The results from this analysis are shown in Figure 5. Focusing first on the associations among variants of the same viruses, SCV^A^ and SCV^B^ were found together in the same individual aphids significantly more than expected by shared chance (*r_p_* = 0.395, 94 d.f., *p* < 0.001). StrV-1^A^ and StrV-1^B^ were also significantly correlated (*r_p_* = 0.729, 94 d.f., *p* < 0.001) among them, but not with StrV-1^C^. Likewise, the three SMoV RNA1 variants were significantly associated among them (*r_p_* ≥ 0.648, 94 d.f., *p* < 0.001). Moving now to the associations among variants of different viruses, only some significant associations were found for the cytorhabdoviruses; most remarkably, SCV^A^ showed a significant association with the three StrV-1 variants (*r_p_* ≥ 0.377, 94 d.f., *p* < 0.001), while SCV^B^ was significantly associated only with StrV-1^A^ and StrV-1^B^ (*r_p_* ≥ 0.458, 94 d.f., *p* < 0.001). No other interspecific significant association was obtained.

### 3.8. Single Aphid Virus Transmission Experiments

**Figure 6:**
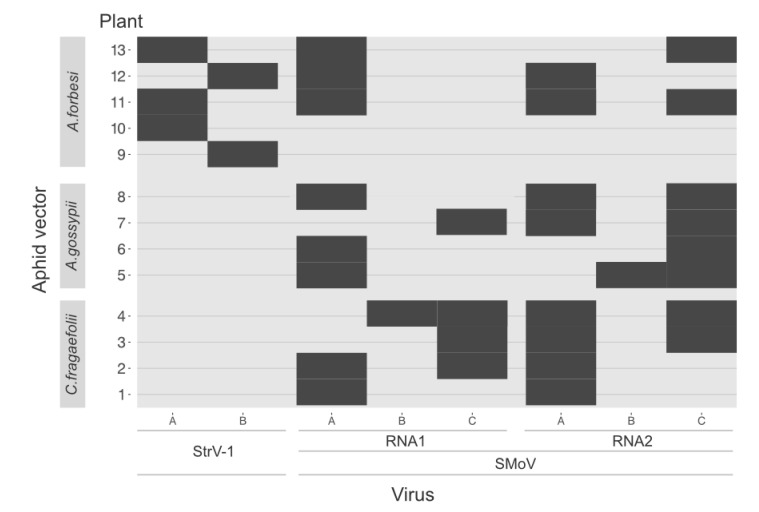
Results of testing of *F. vesca* plants 30 dpi following single aphid transmission with an inoculation access period of 24 h. Black rectangles indicate positive detection.

Wingless adults of *A. forbesi* (*n* = 28), *A. gossypii* (*n* = 30) and *C. fragaefolii* (*n* = 28) were individually transferred from the CRM3 plant onto a leaf of a recipient *F. vesca* plant and left for 24 h. The plants were analyzed for the presence of virus infection at 30 dpi and the positive results are shown in Figure 6.

Levels of successful transmission were 18%, 13% and 14% for *A. forbesi*, *A. gossypii* and *C. fragaefolii*, respectively. There were 12 unique combinations of viruses and their variants out of 13 virus-positive plants (plants 3 and 7 had identical viral infections). Interestingly, there were no cases of SCV transmission by any of the three used aphid species, while StrV-1 was transmitted only by *A. forbesi*. Cases of special interest include plants 9 and 10, where *A. forbesi* transmitted StrV-1, but no SMoV infection was detected.

Mixed infections diagnosed by RT-qPCR were further confirmed by Sanger sequencing of other genomic regions (Table S1).

## 4. Discussion

Error-prone RNA viral polymerases create substantial genetic variability that might feed virus evolution. During the progression of a viral infection, a mutant cloud is formed that encompasses a wide range of closely related entities, usually differing by a few nucleotides [8,42]. Additional genetic variability can be generated throughout the process of recombination, provided multiple genotypes coinfect the same individual cell and interact to produce a chimeric offspring [43,44,45]. Therefore, the frequency at which hosts are co-infected with several viruses, or viral strains of the same virus, is of exceptional interest to plant virologists.

When a CRM3 strawberry plant was subjected to HTS, a complex mixture of coinfecting viruses and genetic variants was found. Firstly, two SCV genotypes coexisted (sharing 84% of nucleotide identity [36]). Secondly, the plant was also infected with three sequence variants of a second cytorhabdovirus, StrV-1 [29]. Thirdly, to add more interest to the picture, a third coinfecting virus was also found, SMoV, which further had a total of six different RNAs—three of each genomic segment. Previously, we found that 26% (11/42) of collected strawberry samples were infected by more than one isolate of StrV-1 [29], which indicated that observed sequence variability and co-infections are not rare events, at least for StrV-1. Correspondingly, we focused on the transmission peculiarities of these three different viral species and their variants.

As initial genomic sequences were derived using HTS, the coverage of some genomes was rather low and there were multiple gaps in SCV^A^, SCV^B^ and StrV-1^C^ assemblies, which were then completed with Sanger sequencing and additional HTS runs [29,36]. In this study, HTS-processed viral amplicons were analyzed for the presence of chimeric sequences, including variant recombinants and defective genomes, which are commonly generated during RNA genome replication [10,39,42]. Such molecules might lead to misinterpretation of the RT-qPCR-based detection of viral variants. No recombination events or chimeric sequences were detected in the current study. It was earlier noted that recombination events in viruses with negative-sense genomes (such as SCV and StrV-1 rhabdoviruses) are rarely observed [46,47] as the viral RNA genome exists as a ribonucleoprotein associated with the nucleocapsid protein, while particular sequence identity and secondary structure requirements should be met [43]. Both SMoV genomic segments were amplified using one set of primers hybridizing in 5′ and 3′ untranslated regions. Despite the extensive description of defective RNAs in tomato black ring virus [48], a member of the same *Secoviridae* family as SMoV, we have not detected such in SMoV. In addition, the genotype-specific RT-qPCR was verified with Sanger sequencing of StrV-1- and SMoV-positive plants after the single aphid transmission.

Successful (88%) graft transmission of SCV, SMoV and StrV-1 and all their variants showed that both the viruses and their variants were able to systemically colonize new recipient hosts, which confirms the absence of strong bottlenecks during this type of transmission. Interestingly, symptomatic infections were observed only in SMoV-positive plants, irrespective of SCV and/or StrV-1 infection.

The original CRM3 strawberry plant was kept for over 20 years [29], and the analyses from the current study did not show the gain or loss of any other virus variants. Generally, for related viruses, such as SCV and StrV-1 or variants of the same species, antagonistic interactions are observed as they compete for the same host resources [5,49]. However, we have not acquired any plants infected with SCV during the current study, so SCV and StrV-1 interactions may be targeted in future studies. A well-known consequence of such antagonistic interactions between strains of the same species is cross-protection, which prevents the establishment of superinfection [13,46]. It could not be determined how the CRM3 plant had originally become infected with the currently observed set of viral variants as follows: either sequentially, i.e., overcoming a cross-protection barrier, or simultaneously all at once, or in a mixed manner. The results of two separate cross-protection experiments with available plants infected with certain either SMoV or StrV-1 variants indicated that the used variants of both viruses did not provide variant-against-variant protection. Future studies might focus on interactions between viral variants from the perspective of replicative fitness, i.e., how the performance of individual viral variants differs depending on the single- or multiple-variant mode of the infection.

Worthy of note is the heterogeneous prevalence of viruses and, in particular, their variants in individual aphids, although each studied aphid population consisted of genetically identical individuals. While both cytorhabdoviruses, due to their propagative mode of transmission, have a much closer relationship with the aphid and must overcome multiple barriers to reach the viruliferous status of the vector, SMoV is transmitted in a non-persistent manner. Nevertheless, the obtained data showed significant differences in virus acquisition for the three tested aphid species regardless of the transmission mode. Quite interesting is the observation that aphid individuals were detected to have only the RNA2 part of the SMoV genome. It should be investigated as to whether they efficiently transmit such incomplete genomes to already SMoV-infected plants, thus increasing the complexity of the existing SMoV infection.

Interestingly, the strawberry aphid *C. fragaefolii*, a well-known strawberry pest, appeared to not be efficient in vectoring StrV-1, as well as the melon aphid *A. gossypii*. The latter is known for its broad range of host plants and transmitted viruses [50]. However, the strawberry root aphid *A. forbesi* showed statistically better virus acquisition than the other two species, which corresponded with higher percentages of rhabdovirus-positive individuals (81% in *A. forbesi* versus 17% and 3% in *C. fragaefolii* and *A. gossypii*, respectively). Interestingly, some genetic variants of the same virus appear to be found in aphids together in a statistically significant manner. Furthermore, most, but not all, variants of the two cytorhabdoviruses were also found together more often than expected by chance. Future studies may focus on understanding the differences in rhabdoviral levels of accumulation among individual aphids.

Single-aphid transmission did not result in high percentages of virus-infected plants. However, it demonstrated the aphids’ ability to simultaneously vector several variants of SMoV. There were general differences in SMoV variant transmission. SMoV RNA1^A^, RNA2^A^ and RNA2^C^ were predominantly found in positive plants, although ∆*C_q_* values for at least RNA1^B^ were higher than for RNA1^A^ in aphids (Figure 2). *A. forbesi* efficiently transmitted StrV-1 in addition to SMoV. Interestingly, all the StrV-1-positive cases contained only a single virus variant.

Based on the cross-protection experiment and single-aphid transmission results, we may speculate that in nature, aphids transmit either multiple viral variants simultaneously or introduce new variants into already virus-infected plants. For example, in the case of CTV, it was suggested that the interaction between different coinfecting variants influences transmission outcomes, including complementation of the transmission-deficient isolates [15,51]. The other factor leading to the establishment of infection with multiple variants of the same virus in nature might be the subsequent colonization of nearby plants by multiple aphids, each vectoring one or more viral variants.

## 5. Conclusions

Individual aphids colonizing virus-infected plants acquire viruses and their variants in a different manner. Single-aphid transmission experiments led to the simultaneous inoculation of multiple SMoV variants. Studied SMoV (and StrV-1) variants did not cross-protect infected plants against each other.

## Figures and Tables

**Table 1:** Summarized percentages of positive aphid individuals from Figure 4.

Aphid Species	Virus and Variant										
	SCV		StrV-1			SMoV					
	A	B	A	B	C	RNA1^A^	RNA1^B^	RNA1^C^	RNA2^A^	RNA2^B^	RNA2^C^
*A. forbesi*	39	65	77	71	16	58	77	77	97	100	100
*A. gossypii*	3	0	0	3	0	100	100	97	100	100	90
*C. fragaefolii*	0	17	0	0	0	43	63	66	97	97	86

## Data Availability

Data generated during the study were deposited at the NCBI repository under accession numbers SAMN27687906-8, OK181865.1, MN420510.1 and ON756034-7.

## Supplementary Materials

The following supporting information can be downloaded at: https://www.mdpi.com/article/10.3390/v14071362/s1, Figure S1: Scheme of strand-specific cDNA synthesis; Figure S2: Agarose gel electrophoresis of the amplified fragments of SCV, SMoV and StrV-1; Table S1: List of primers used in the study; Table S2: Overview of mappings of HTS reads on the viral references; Table S3: Detailed listing of detected nucleotide variation in SCV, SMoV and StrV-1; Table S4: Counts of variable positions and their effect on encoded proteins; Table S5: Overview of ∆*C_q_* values for SCV, SMoV, StrV-1 and their variants in individual aphid adults. References [37,38] are cited in the Supplementary Materials.
