# Supplementary material for: Transmission of Diverse Variants of Strawberry Viruses Is Governed by a Vector Species
Source: Viruses. 2022 Jun 23;14(7):1362. doi: 10.3390/v14071362 (PMC9316375; doi:10.3390/v14071362)
Supplement: Supplementary file 1 [file viruses-14-01362-s001.zip › Figure S2.pdf]

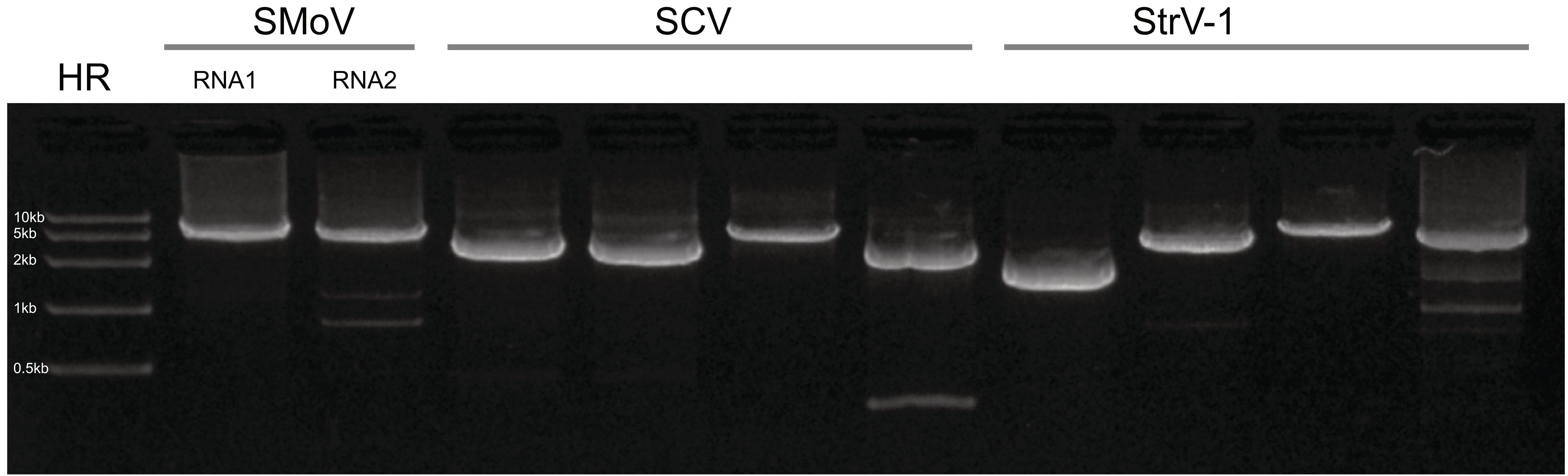

Figure S2 Agarose gel electrophoresis of the amplified fragments of strawberry crinkle virus (SCV), strawberry mottle virus (SMoV) and strawberry virus 1 (StrV-1), HR - HighRange ladder (0.5 - 10 kb).
