# Supplementary figures and images for: Transmission of Diverse Variants of Strawberry Viruses Is Governed by a Vector Species
Source: Viruses. 2022 Jun 23;14(7):1362. doi: 10.3390/v14071362 (PMC9316375; doi:10.3390/v14071362)

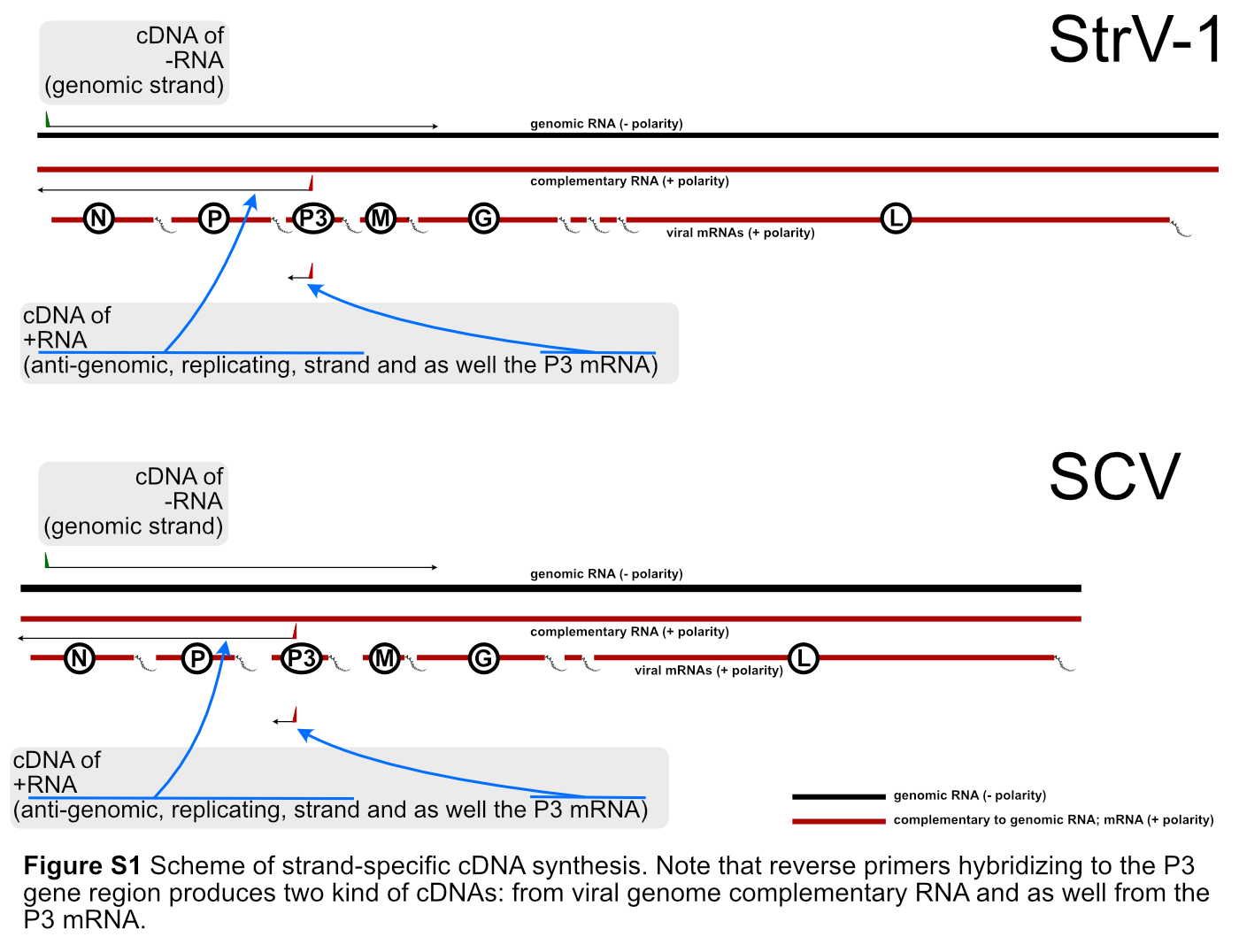

Supplement: Supplementary file 1 [file viruses-14-01362-s001.zip › Figure S1.png]
